# μ-Succinato-κ^2^ *O* ^1^:*O* ^4^-bis[(2,2′-bipyridine-*κ* ^2^ *N*,*N*′)copper(II)] succinate dodeca­hydrate

**DOI:** 10.1107/S160053680904224X

**Published:** 2009-10-23

**Authors:** Jian-Li Lin, Wei Xu

**Affiliations:** a State Key Laboratory Base of Novel Functional Materials and Preparation Science, Center of Applied Solid State Chemistry Research, Ningbo University, Ningbo 315211, People’s Republic of China

## Abstract

In the title compound, [Cu_2_(C_4_H_4_O_4_)(C_10_H_8_N_2_)_4_]C_4_H_4_O_4_·12H_2_O, C_10_H_8_N_2_), the centrosymmetic dinuclear cations, succinate anions and water mol­ecules are hydrogen bonded into layers parallel to (010). The Cu atom is square-pyramidally coordinated by one atom of the succinato ligand and four N atoms of two 2,2′-bipyridine ligands. The 12 water mol­ecules form a new type of water cluster.

## Related literature

For metal-organic coordination polymers, see: Batten & Robson (1998 ▶); Rao *et al.* (2004 ▶); Zheng *et al.* (2004 ▶). The configuration of water clusters depends on the environment of the host, see: Wei *et al.* (2006 ▶). 
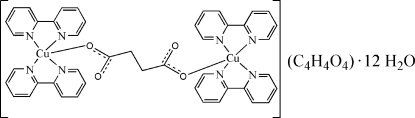


## Experimental

### 

#### Crystal data

[Cu_2_(C_4_H_4_O_4_)(C_10_H_8_N_2_)_4_]C_4_H_4_O_4_·12H_2_O
*M* *_r_* = 1200.15
Triclinic, 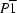

*a* = 10.502 (2) Å
*b* = 10.764 (2) Å
*c* = 12.892 (3) Å
α = 77.21 (3)°
β = 77.99 (3)°
γ = 79.85 (3)°
*V* = 1377.1 (5) Å^3^
*Z* = 1
Mo *K*α radiation
μ = 0.85 mm^−1^
*T* = 295 K
0.34 × 0.27 × 0.19 mm

#### Data collection

Bruker P4 diffractometer
Absorption correction: ψ scan (*XSCANS*, Siemens, 1996 ▶) *T*_min_ = 0.750, *T*_max_ = 0.844
5721 measured reflections
4853 independent reflections
3856 reflections with *I* > 2σ(*I*)
*R*_int_ = 0.025
3 standard reflections every 97 reflections intensity decay: none

#### Refinement

*R*[*F* ^2^ > 2σ(*F* ^2^)] = 0.053
*wR*(*F* ^2^) = 0.145
*S* = 1.07
4853 reflections
353 parameters
18 restraints
H-atom parameters constrained
Δρ_max_ = 1.03 e Å^−3^
Δρ_min_ = −1.58 e Å^−3^

### 

Data collection: *XSCANS* (Siemens, 1996 ▶); cell refinement: *XSCANS*; data reduction: *XSCANS*; program(s) used to solve structure: *SHELXS97* (Sheldrick, 2008 ▶); program(s) used to refine structure: *SHELXL97* (Sheldrick, 2008 ▶); molecular graphics: *SHELXTL* (Sheldrick, 2008 ▶); software used to prepare material for publication: *SHELXL97*.

## Supplementary Material

Crystal structure: contains datablocks global, I. DOI: 10.1107/S160053680904224X/ng2666sup1.cif

Structure factors: contains datablocks I. DOI: 10.1107/S160053680904224X/ng2666Isup2.hkl

Additional supplementary materials:  crystallographic information; 3D view; checkCIF report

## Figures and Tables

**Table 1:** Hydrogen-bond geometry (Å, °)

*D*—H⋯*A*	*D*—H	H⋯*A*	*D*⋯*A*	*D*—H⋯*A*
O5—H5*B*⋯O10^i^	0.85	1.96	2.750 (6)	154
O5—H5*C*⋯O9	0.85	1.90	2.739 (6)	169
O6—H6*B*⋯O5	0.84	1.99	2.820 (6)	168
O6—H6*C*⋯O5^ii^	0.86	2.01	2.847 (7)	167
O7—H7*B*⋯O6^iii^	0.85	2.01	2.755 (5)	146
O7—H7*C*⋯O8	0.84	1.98	2.820 (5)	175
O8—H8*B*⋯O2^iv^	0.85	1.95	2.795 (5)	177
O8—H8*C*⋯O3	0.85	1.85	2.682 (5)	169
O9—H9*B*⋯O8^iii^	0.84	2.02	2.850 (5)	172
O9—H9*C*⋯O4	0.86	1.85	2.704 (6)	176
O10—H10*B*⋯O7^v^	0.84	2.09	2.877 (7)	156
O10—H10*C*⋯O4	0.85	1.99	2.758 (7)	149

Symmetry codes: (i) 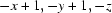; (ii) 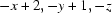; (iii) 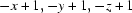; (iv) 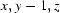; (v) 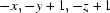.

**Fig. 1.**
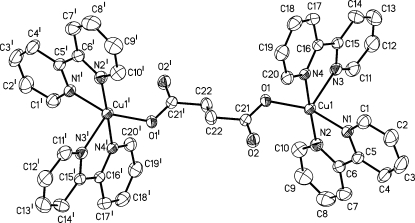
ORTEP view of the title compound. The dispalcement ellipsoids are drawn at 40% probability level. [Symmetry code: (I) -x + 1, -y + 2, -z + 1]

**Fig. 2.**
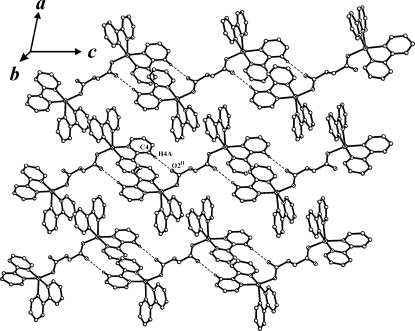
The two-dimensional layer for the supramolecular assembly of the complex cations. [Symmetry codes: (II) -x, -y + 2, -z + 2].

**Fig. 3.**
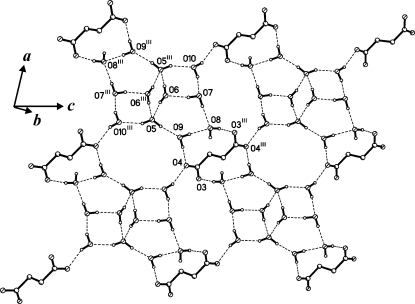
The two-dimensional water-succinate framework parallel to (010).

## supplementary crystallographic information

### Comment

In the past decade, metal-organic coordination polymers have attracted considerable interest due to their potential applications and intriguing architectures (Batten & Robson, 1998). The saturated aliphatic dicarboxylate ligands, which exhibit conformational and coordination versatility due to single-bonded carbon chains, are also an attractive choice and considered as important flexible spacer ligand (Rao, *et al.*, 2004). As one of the lower members in the α,ω-dicarboxylate family, the succinate anions play a special role. Under ambient conditions, the linkage of transition metal cations by succinate anions may lead to linear polymeric chains, two-dimensional open networks and three-dimensional framework coordination polymers (Zheng, *et al.*, 2004). Some metal-organic coordination polymers are open-frameworked and there are guest species occluded by open-framework host in the structures. Water molecules are the commonly encountered guest species, and they usually play an important role in the stabilization of the host, on the other hand, the configuration of water clusters depends on the surrounding environment of the host (Wei, *et al.*, 2006). A variety of water clusters observed in a number of hosts have been structurally characterized to help us gain insight into the nature of water-water interactions. Herein, we report the presence of a new type (H_2_O)_12_ water cluster in the structure of succinato bridged dinuclear complex [Cu_2_(bpy)_4_(C_4_H_4_O_4_)](C_4_H_4_O_4_).12H_2_O.

The title compound consists of succinato bridged dinuclear [Cu_2_(bpy)_4_(C_4_H_4_O_4_)]^2+^ complex cations, succinate anions and crystal water molecules. As illustrated in Fig. 1, Cu^2+^ in the complex cations are each square pyramidally coordinated by one O atom of the succinato ligand and four N atoms of two bpy ligands with the N3 atom at the apical position (*d*(Cu—O) = 1.981 (3) Å; equatorial d(Cu—N) = 2.000 (4)–2.033 (3) Å; axial d(Cu—N) = 2.172 (3) Å). The Cu atom is shift by 0.179 (2) from the equatorial plane through N1, N2, N4 and O4 atoms towards the apical N3 atom. The succinato group bis-bidentately bridges two Cu ions to form the dinuclear complex cation. Such bridging succinato ligand and the noncoordinating succinate anion are also exhibit *trans* conformation with the backbone C atoms in a plane. The dehedral angles between the two pyridine rings are twisted by 8.34° and 10.08° in the two different bpy ligands. The complex cations are forced to be aligned in such ways that the symmetry related bpy ligands are orientated parallelly and face the opposite directions with the interplanar distanceds varying from 3.533 Å to 3.541 Å. Obviously, sucn π-π stacking interactions are responsible for the supramolecular assembly of the complex molecules into two-dimensional layers parallel to (010). The resulting complex cationic layers are found to be stabilized by weak *C*(bpy)-*H*···*O*(carboxylate) hydrogen bonds with d(H4A···O2^II^) = 2.47 Å (II: -*x*, -*y* + 2, -*z* + 2) (Fig. 2).

The most interesting feature of the solid-state structure of the title complex is the hydrogen-bonding interactions of the water molecules and the succinate anion, in which twelve water molecules form a (H_2_O)_12_ cluster associated by O—H···O hydrogen bonds (as shown in Fig 3). The geometric parameters of the clusters are summarized in Tables 1. The O···O distances range from 2.738 (6)–2.876 (7) Å and the angles of the O—H···O are vary from 145° to 175°. Interestingly, such (H_2_O)_12_ water clusters are farther hydrogen bond interacting with succinate anions to complete two-dimensional layers parallel to (010) with (*d*(O···O) = 2.682 (5)–2.758 (7) Å; <O—H···O = 149–176°).

### Experimental

Addition of 10 ml CH_3_OH containing 0.324 g (2.08 mmol) 2,2'-bipyridine (bpy) to an aqueous solution of 0.171 g (1.00 mmol) CuCl_2_.2H_2_O in 10 ml H_2_O gave a blue solution, then added 0.182 g (1.00 mmol) succinic acid to the mixture. The mixture was further stirred vigorously, and the resulting blue solution was adjusted with NaOH to pH = 8.3 and allowed to stand at room temperature. After two weeks, a small amount of blue block crystals had grown.

### Refinement

H atoms bonded to C atoms were palced in geometrically calculated position and were refined using a riding model, with *U*_iso_(H) = 1.2 *U*_eq_(C). H atoms attached to O atoms were found in a difference Fourier synthesis and were refined using a riding model.

### Crystal data

**Table:**

[Cu_2_(C_4_H_4_O_4_)(C_10_H_8_N_2_)_4_]C_4_H_4_O_4_·12H_2_O	*Z* = 1
*M**_r_* = 1200.15	*F*(000) = 626
Triclinic, *P*1	*D*_x_ = 1.447 Mg m^−^^3^
Hall symbol: -P 1	Mo *K*α radiation, λ = 0.71073 Å
*a* = 10.502 (2) Å	Cell parameters from 25 reflections
*b* = 10.764 (2) Å	θ = 5.0–12.5°
*c* = 12.892 (3) Å	µ = 0.85 mm^−^^1^
α = 77.21 (3)°	*T* = 295 K
β = 77.99 (3)°	Block, blue
γ = 79.85 (3)°	0.34 × 0.27 × 0.19 mm
*V* = 1377.1 (5) Å^3^	

### Data collection

**Table:**

Bruker P4 diffractometer	3856 reflections with *I* > 2σ(*I*)
Radiation source: fine-focus sealed tube	*R*_int_ = 0.025
graphite	θ_max_ = 25.0°, θ_min_ = 1.7°
θ/2θ scans	*h* = −1→12
Absorption correction: ψ scan *XSCANS*	*k* = −12→12
*T*_min_ = 0.750, *T*_max_ = 0.844	*l* = −15→15
5721 measured reflections	3 standard reflections every 97 reflections
4853 independent reflections	intensity decay: none

### Refinement

**Table:**

Refinement on *F*^2^	Primary atom site location: structure-invariant direct methods
Least-squares matrix: full	Secondary atom site location: difference Fourier map
*R*[*F*^2^ > 2σ(*F*^2^)] = 0.053	Hydrogen site location: inferred from neighbouring sites
*wR*(*F*^2^) = 0.145	H-atom parameters constrained
*S* = 1.07	*w* = 1/[σ^2^(*F*_o_^2^) + (0.0531*P*)^2^ + 2.9966*P*] where *P* = (*F*_o_^2^ + 2*F*_c_^2^)/3
4853 reflections	(Δ/σ)_max_ = 0.001
353 parameters	Δρ_max_ = 1.03 e Å^−^^3^
18 restraints	Δρ_min_ = −1.58 e Å^−^^3^

### Special details

**Table:**

Geometry. All e.s.d.'s (except the e.s.d. in the dihedral angle between two l.s. planes) are estimated using the full covariance matrix. The cell e.s.d.'s are taken into account individually in the estimation of e.s.d.'s in distances, angles and torsion angles; correlations between e.s.d.'s in cell parameters are only used when they are defined by crystal symmetry. An approximate (isotropic) treatment of cell e.s.d.'s is used for estimating e.s.d.'s involving l.s. planes.
Refinement. Refinement of *F*^2^ against ALL reflections. The weighted *R*-factor *wR* and goodness of fit *S* are based on *F*^2^, conventional *R*-factors *R* are based on *F*, with *F* set to zero for negative *F*^2^. The threshold expression of *F*^2^ > σ(*F*^2^) is used only for calculating *R*-factors(gt) *etc*. and is not relevant to the choice of reflections for refinement. *R*-factors based on *F*^2^ are statistically about twice as large as those based on *F*, and *R*- factors based on ALL data will be even larger.

### Fractional atomic coordinates and isotropic or equivalent isotropic displacement parameters (Å^2^)

**Table:**

	*x*	*y*	*z*	*U*_iso_*/*U*_eq_	
Cu1	0.74197 (5)	0.97679 (5)	0.74602 (4)	0.03307 (17)	
N1	0.7538 (3)	1.0058 (3)	0.8941 (3)	0.0375 (8)	
N2	0.6286 (4)	0.8488 (3)	0.8385 (3)	0.0393 (8)	
N3	0.9308 (3)	0.8594 (3)	0.7123 (3)	0.0385 (8)	
N4	0.8548 (3)	1.1107 (3)	0.6606 (2)	0.0328 (7)	
O1	0.6673 (3)	0.9791 (3)	0.6165 (2)	0.0370 (7)	
O2	0.5113 (3)	1.1205 (3)	0.6850 (2)	0.0479 (8)	
O3	0.3066 (4)	0.3885 (4)	0.4609 (3)	0.0632 (10)	
O4	0.4199 (4)	0.4917 (4)	0.3143 (3)	0.0719 (11)	
O5	0.7964 (4)	0.5153 (5)	0.0060 (3)	0.0875 (14)	
O6	0.9811 (4)	0.6789 (4)	−0.0001 (3)	0.0816 (12)	
O7	0.0176 (4)	0.3001 (5)	0.7912 (3)	0.0787 (12)	
O8	0.2760 (3)	0.2866 (3)	0.6727 (3)	0.0581 (9)	
O9	0.6523 (4)	0.5599 (4)	0.2004 (3)	0.0639 (10)	
O10	0.2260 (5)	0.5285 (5)	0.1913 (4)	0.0970 (16)	
C1	0.8295 (5)	1.0819 (5)	0.9166 (4)	0.0481 (11)	
H1A	0.8774	1.1339	0.8600	0.058*	
C2	0.8378 (6)	1.0848 (5)	1.0216 (4)	0.0603 (14)	
H2A	0.8915	1.1373	1.0353	0.072*	
C3	0.7667 (6)	1.0101 (6)	1.1047 (4)	0.0670 (16)	
H3A	0.7710	1.0117	1.1757	0.080*	
C4	0.6883 (6)	0.9320 (5)	1.0835 (4)	0.0559 (13)	
H4A	0.6389	0.8808	1.1397	0.067*	
C5	0.6844 (4)	0.9312 (4)	0.9766 (3)	0.0380 (10)	
C6	0.6070 (4)	0.8479 (4)	0.9453 (3)	0.0385 (10)	
C7	0.5218 (5)	0.7736 (5)	1.0173 (4)	0.0535 (13)	
H7A	0.5069	0.7758	1.0905	0.064*	
C8	0.4584 (6)	0.6953 (5)	0.9794 (5)	0.0652 (15)	
H8A	0.3998	0.6445	1.0267	0.078*	
C9	0.4830 (6)	0.6936 (5)	0.8710 (5)	0.0645 (15)	
H9A	0.4423	0.6405	0.8441	0.077*	
C10	0.5681 (5)	0.7709 (5)	0.8026 (4)	0.0524 (12)	
H10A	0.5844	0.7694	0.7291	0.063*	
C11	0.9635 (5)	0.7332 (5)	0.7468 (4)	0.0522 (12)	
H11A	0.8972	0.6837	0.7815	0.063*	
C12	1.0915 (6)	0.6737 (5)	0.7330 (4)	0.0642 (16)	
H12A	1.1114	0.5860	0.7585	0.077*	
C13	1.1884 (6)	0.7463 (6)	0.6812 (5)	0.0660 (16)	
H13A	1.2758	0.7087	0.6721	0.079*	
C14	1.1561 (5)	0.8762 (5)	0.6421 (4)	0.0558 (13)	
H14A	1.2210	0.9262	0.6046	0.067*	
C15	1.0263 (4)	0.9304 (4)	0.6595 (3)	0.0373 (10)	
C16	0.9817 (4)	1.0698 (4)	0.6231 (3)	0.0346 (9)	
C17	1.0628 (4)	1.1533 (5)	0.5547 (3)	0.0443 (11)	
H17A	1.1495	1.1236	0.5281	0.053*	
C18	1.0132 (5)	1.2814 (5)	0.5267 (4)	0.0517 (12)	
H18A	1.0666	1.3389	0.4814	0.062*	
C19	0.8851 (5)	1.3235 (5)	0.5658 (4)	0.0494 (12)	
H19A	0.8507	1.4096	0.5480	0.059*	
C20	0.8082 (5)	1.2362 (4)	0.6321 (3)	0.0420 (10)	
H20A	0.7210	1.2646	0.6582	0.050*	
C21	0.5588 (4)	1.0531 (4)	0.6155 (3)	0.0334 (9)	
C22	0.4878 (5)	1.0601 (4)	0.5229 (4)	0.0428 (10)	
H22A	0.3941	1.0796	0.5479	0.051*	
H22B	0.5142	1.1306	0.4655	0.051*	
C23	0.4059 (5)	0.4377 (4)	0.4120 (4)	0.0452 (11)	
C24	0.5143 (5)	0.4410 (4)	0.4746 (4)	0.0441 (11)	
H24A	0.5201	0.3644	0.5306	0.053*	
H24B	0.5981	0.4415	0.4259	0.053*	
H5B	0.7666	0.4960	−0.0436	0.080*	
H5C	0.7444	0.5361	0.0614	0.079*	
H6B	0.9347	0.6242	−0.0038	0.072*	
H6C	1.0555	0.6304	−0.0039	0.075*	
H7B	0.0280	0.3367	0.8405	0.071*	
H7C	0.0944	0.3014	0.7554	0.072*	
H8B	0.3482	0.2377	0.6775	0.073*	
H8C	0.2770	0.3144	0.6058	0.060*	
H9B	0.6769	0.5981	0.2414	0.054*	
H9C	0.5793	0.5400	0.2387	0.061*	
H10B	0.1514	0.5652	0.2154	0.092*	
H10C	0.2638	0.5022	0.2459	0.094*	

### Atomic displacement parameters (Å^2^)

**Table:**

	*U*^11^	*U*^22^	*U*^33^	*U*^12^	*U*^13^	*U*^23^
Cu1	0.0336 (3)	0.0362 (3)	0.0252 (3)	0.0014 (2)	−0.00311 (19)	−0.00379 (19)
N1	0.039 (2)	0.043 (2)	0.0283 (17)	0.0003 (16)	−0.0056 (15)	−0.0060 (15)
N2	0.041 (2)	0.0361 (19)	0.0363 (19)	0.0005 (16)	−0.0058 (16)	−0.0032 (15)
N3	0.041 (2)	0.0392 (19)	0.0289 (17)	0.0054 (16)	−0.0042 (15)	−0.0024 (14)
N4	0.0351 (19)	0.0361 (18)	0.0243 (16)	−0.0008 (15)	−0.0037 (14)	−0.0043 (13)
O1	0.0312 (15)	0.0495 (17)	0.0308 (14)	0.0045 (13)	−0.0103 (12)	−0.0118 (12)
O2	0.0494 (19)	0.0567 (19)	0.0376 (16)	0.0090 (15)	−0.0112 (14)	−0.0185 (15)
O3	0.059 (2)	0.076 (3)	0.051 (2)	−0.014 (2)	−0.0167 (18)	0.0055 (18)
O4	0.068 (3)	0.111 (3)	0.0383 (19)	−0.023 (2)	−0.0130 (17)	−0.007 (2)
O5	0.066 (3)	0.141 (4)	0.066 (3)	−0.011 (3)	−0.002 (2)	−0.053 (3)
O6	0.088 (3)	0.095 (3)	0.068 (3)	−0.020 (3)	−0.005 (2)	−0.030 (2)
O7	0.060 (2)	0.122 (4)	0.057 (2)	−0.017 (2)	0.0023 (19)	−0.032 (2)
O8	0.048 (2)	0.073 (2)	0.0435 (18)	0.0064 (17)	−0.0043 (15)	−0.0068 (16)
O9	0.065 (2)	0.078 (3)	0.0459 (19)	−0.005 (2)	−0.0001 (17)	−0.0187 (18)
O10	0.104 (4)	0.114 (4)	0.080 (3)	0.016 (3)	−0.048 (3)	−0.027 (3)
C1	0.052 (3)	0.055 (3)	0.040 (2)	−0.010 (2)	−0.007 (2)	−0.012 (2)
C2	0.069 (4)	0.068 (3)	0.052 (3)	−0.007 (3)	−0.020 (3)	−0.023 (3)
C3	0.087 (4)	0.081 (4)	0.035 (3)	−0.003 (3)	−0.018 (3)	−0.016 (3)
C4	0.068 (3)	0.064 (3)	0.030 (2)	−0.005 (3)	−0.006 (2)	−0.002 (2)
C5	0.039 (2)	0.039 (2)	0.029 (2)	0.0052 (18)	−0.0038 (18)	−0.0028 (17)
C6	0.036 (2)	0.034 (2)	0.036 (2)	0.0076 (18)	−0.0032 (18)	0.0004 (17)
C7	0.051 (3)	0.048 (3)	0.047 (3)	0.000 (2)	0.001 (2)	0.006 (2)
C8	0.060 (3)	0.048 (3)	0.074 (4)	−0.015 (3)	0.006 (3)	0.008 (3)
C9	0.065 (4)	0.049 (3)	0.078 (4)	−0.016 (3)	−0.009 (3)	−0.008 (3)
C10	0.059 (3)	0.045 (3)	0.053 (3)	−0.008 (2)	−0.008 (2)	−0.009 (2)
C11	0.059 (3)	0.043 (3)	0.043 (3)	0.006 (2)	−0.005 (2)	0.000 (2)
C12	0.070 (4)	0.055 (3)	0.054 (3)	0.027 (3)	−0.012 (3)	−0.009 (2)
C13	0.052 (3)	0.073 (4)	0.067 (3)	0.028 (3)	−0.012 (3)	−0.027 (3)
C14	0.038 (3)	0.067 (3)	0.057 (3)	0.009 (2)	0.000 (2)	−0.021 (3)
C15	0.035 (2)	0.049 (2)	0.027 (2)	0.0061 (19)	−0.0053 (17)	−0.0140 (18)
C16	0.032 (2)	0.048 (2)	0.0248 (19)	−0.0017 (18)	−0.0056 (16)	−0.0114 (17)
C17	0.037 (2)	0.058 (3)	0.035 (2)	−0.008 (2)	0.0010 (19)	−0.010 (2)
C18	0.061 (3)	0.056 (3)	0.039 (2)	−0.023 (3)	−0.005 (2)	−0.003 (2)
C19	0.061 (3)	0.039 (2)	0.046 (3)	−0.007 (2)	−0.012 (2)	−0.002 (2)
C20	0.043 (3)	0.040 (2)	0.039 (2)	0.0033 (19)	−0.0093 (19)	−0.0043 (19)
C21	0.035 (2)	0.038 (2)	0.0276 (19)	−0.0058 (18)	−0.0065 (17)	−0.0046 (17)
C22	0.042 (3)	0.045 (2)	0.043 (2)	0.009 (2)	−0.017 (2)	−0.014 (2)
C23	0.053 (3)	0.046 (3)	0.037 (2)	0.006 (2)	−0.013 (2)	−0.015 (2)
C24	0.044 (3)	0.044 (2)	0.044 (2)	0.009 (2)	−0.012 (2)	−0.015 (2)

### Geometric parameters (Å, °)

**Table:**

Cu1—O1	1.981 (3)	C4—C5	1.389 (6)
Cu1—N2	2.000 (4)	C4—H4A	0.9300
Cu1—N4	2.013 (3)	C5—C6	1.476 (7)
Cu1—N1	2.033 (3)	C6—C7	1.371 (6)
Cu1—N3	2.172 (3)	C7—C8	1.381 (8)
Cu1—O2	2.795 (3)	C7—H7A	0.9300
N1—C5	1.347 (5)	C8—C9	1.371 (8)
N1—C1	1.349 (6)	C8—H8A	0.9300
N2—C10	1.343 (6)	C9—C10	1.370 (7)
N2—C6	1.346 (5)	C9—H9A	0.9300
N3—C11	1.340 (6)	C10—H10A	0.9300
N3—C15	1.345 (6)	C11—C12	1.376 (7)
N4—C20	1.349 (5)	C11—H11A	0.9300
N4—C16	1.349 (5)	C12—C13	1.363 (8)
O1—C21	1.271 (5)	C12—H12A	0.9300
O2—C21	1.246 (5)	C13—C14	1.385 (8)
O3—C23	1.240 (6)	C13—H13A	0.9300
O4—C23	1.254 (6)	C14—C15	1.377 (6)
O5—H5B	0.8457	C14—H14A	0.9300
O5—H5C	0.8510	C15—C16	1.491 (6)
O6—H6B	0.8421	C16—C17	1.385 (6)
O6—H6C	0.8584	C17—C18	1.381 (7)
O7—H7B	0.8517	C17—H17A	0.9300
O7—H7C	0.8424	C18—C19	1.368 (7)
O8—H8B	0.8491	C18—H18A	0.9300
O8—H8C	0.8459	C19—C20	1.375 (6)
O9—H9B	0.8404	C19—H19A	0.9300
O9—H9C	0.8548	C20—H20A	0.9300
O10—H10B	0.8424	C21—C22	1.516 (6)
O10—H10C	0.8505	C22—C22^i^	1.499 (8)
C1—C2	1.381 (7)	C22—H22A	0.9700
C1—H1A	0.9300	C22—H22B	0.9700
C2—C3	1.360 (8)	C23—C24	1.536 (6)
C2—H2A	0.9300	C24—C24^ii^	1.511 (8)
C3—C4	1.378 (8)	C24—H24A	0.9700
C3—H3A	0.9300	C24—H24B	0.9700
			
O1—Cu1—N2	92.51 (14)	C9—C8—H8A	120.5
O1—Cu1—N4	90.27 (13)	C7—C8—H8A	120.5
N2—Cu1—N4	176.56 (14)	C10—C9—C8	119.4 (6)
O1—Cu1—N1	159.82 (13)	C10—C9—H9A	120.3
N2—Cu1—N1	80.65 (15)	C8—C9—H9A	120.3
N4—Cu1—N1	96.06 (14)	N2—C10—C9	121.9 (5)
O1—Cu1—N3	101.12 (12)	N2—C10—H10A	119.1
N2—Cu1—N3	102.72 (14)	C9—C10—H10A	119.1
N4—Cu1—N3	78.70 (13)	N3—C11—C12	122.6 (5)
N1—Cu1—N3	98.90 (14)	N3—C11—H11A	118.7
O1—Cu1—O2	51.85 (10)	C12—C11—H11A	118.7
N2—Cu1—O2	86.73 (13)	C13—C12—C11	118.5 (5)
N4—Cu1—O2	93.40 (12)	C13—C12—H12A	120.7
N1—Cu1—O2	108.49 (12)	C11—C12—H12A	120.7
N3—Cu1—O2	152.18 (11)	C12—C13—C14	119.6 (5)
C5—N1—C1	118.7 (4)	C12—C13—H13A	120.2
C5—N1—Cu1	113.8 (3)	C14—C13—H13A	120.2
C1—N1—Cu1	127.3 (3)	C15—C14—C13	119.0 (5)
C10—N2—C6	118.7 (4)	C15—C14—H14A	120.5
C10—N2—Cu1	125.7 (3)	C13—C14—H14A	120.5
C6—N2—Cu1	115.6 (3)	N3—C15—C14	121.4 (4)
C11—N3—C15	118.7 (4)	N3—C15—C16	115.4 (4)
C11—N3—Cu1	128.5 (3)	C14—C15—C16	123.2 (4)
C15—N3—Cu1	112.3 (3)	N4—C16—C17	121.3 (4)
C20—N4—C16	118.7 (4)	N4—C16—C15	115.5 (4)
C20—N4—Cu1	123.6 (3)	C17—C16—C15	123.2 (4)
C16—N4—Cu1	117.4 (3)	C18—C17—C16	119.0 (4)
C21—O1—Cu1	111.5 (2)	C18—C17—H17A	120.5
C21—O2—Cu1	73.5 (2)	C16—C17—H17A	120.5
H5B—O5—H5C	120.3	C19—C18—C17	119.8 (4)
H6B—O6—H6C	97.8	C19—C18—H18A	120.1
H7B—O7—H7C	97.0	C17—C18—H18A	120.1
H8B—O8—H8C	105.8	C18—C19—C20	118.8 (4)
H9B—O9—H9C	100.3	C18—C19—H19A	120.6
H10B—O10—H10C	104.9	C20—C19—H19A	120.6
N1—C1—C2	121.8 (5)	N4—C20—C19	122.3 (4)
N1—C1—H1A	119.1	N4—C20—H20A	118.8
C2—C1—H1A	119.1	C19—C20—H20A	118.8
C3—C2—C1	119.3 (5)	O2—C21—O1	123.1 (4)
C3—C2—H2A	120.4	O2—C21—C22	119.7 (4)
C1—C2—H2A	120.4	O1—C21—C22	117.2 (3)
C2—C3—C4	119.9 (5)	C22^i^—C22—C21	114.7 (4)
C2—C3—H3A	120.1	C22^i^—C22—H22A	108.6
C4—C3—H3A	120.1	C21—C22—H22A	108.6
C3—C4—C5	118.7 (5)	C22^i^—C22—H22B	108.6
C3—C4—H4A	120.6	C21—C22—H22B	108.6
C5—C4—H4A	120.6	H22A—C22—H22B	107.6
N1—C5—C4	121.6 (4)	O3—C23—O4	123.4 (5)
N1—C5—C6	115.5 (4)	O3—C23—C24	119.0 (4)
C4—C5—C6	122.9 (4)	O4—C23—C24	117.5 (5)
N2—C6—C7	121.9 (5)	C24^ii^—C24—C23	110.8 (4)
N2—C6—C5	114.1 (4)	C24^ii^—C24—H24A	109.5
C7—C6—C5	124.0 (4)	C23—C24—H24A	109.5
C6—C7—C8	119.1 (5)	C24^ii^—C24—H24B	109.5
C6—C7—H7A	120.5	C23—C24—H24B	109.5
C8—C7—H7A	120.5	H24A—C24—H24B	108.1
C9—C8—C7	119.0 (5)		
			
C23—C24—C24^ii^—C23^ii^	180.000 (1)		

Symmetry codes: (i) −*x*+1, −*y*+2, −*z*+1; (ii) −*x*+1, −*y*+1, −*z*+1.

### Hydrogen-bond geometry (Å, °)

**Table:**

*D*—H···*A*	*D*—H	H···*A*	*D*···*A*	*D*—H···*A*
O5—H5B···O10^iii^	0.85	1.96	2.750 (6)	154
O5—H5C···O9	0.85	1.90	2.739 (6)	169
O6—H6B···O5	0.84	1.99	2.820 (6)	168
O6—H6C···O5^iv^	0.86	2.01	2.847 (7)	167
O7—H7B···O6^ii^	0.85	2.01	2.755 (5)	146
O7—H7C···O8	0.84	1.98	2.820 (5)	175
O8—H8B···O2^v^	0.85	1.95	2.795 (5)	177
O8—H8C···O3	0.85	1.85	2.682 (5)	169
O9—H9B···O8^ii^	0.84	2.02	2.850 (5)	172
O9—H9C···O4	0.86	1.85	2.704 (6)	176
O10—H10B···O7^vi^	0.84	2.09	2.877 (7)	156
O10—H10C···O4	0.85	1.99	2.758 (7)	149

Symmetry codes: (iii) −*x*+1, −*y*+1, −*z*;  (iv) −*x*+2, −*y*+1, −*z*; (ii) −*x*+1, −*y*+1, −*z*+1; (v) *x*, *y*−1, *z*; (vi) −*x*, −*y*+1, −*z*+1.
